# Meta-Analysis of Adipose Tissue Derived Cell-Based Therapy for the Treatment of Knee Osteoarthritis

**DOI:** 10.3390/cells10061365

**Published:** 2021-06-01

**Authors:** Nikhil Agarwal, Christopher Mak, Christine Bojanic, Kendrick To, Wasim Khan

**Affiliations:** 1 MBChB Office, University of Aberdeen College of Life Sciences and Medicine, Foresterhill Rd, Aberdeen AB25 2ZD, UK; nikagarwal@live.co.uk; 2 Division of Trauma & Orthopaedic Surgery, Addenbrooke’s Hospital, University of Cambridge, Cambridge CB2 0QQ, UK; chcm2@cam.ac.uk (C.M.); cbojanic@doctors.org.uk (C.B.); kendrick.to@doctors.org.uk (K.T.)

**Keywords:** osteoarthritis, degenerative changes, knee, adipose tissue, mesenchymal stem cells, stromal vascular factor

## Abstract

Osteoarthritis (OA) is a degenerative disorder associated with cartilage loss and is a leading cause of disability around the world. In old age, the capacity of cartilage to regenerate is diminished. With an aging population, the burden of OA is set to rise. Currently, there is no definitive treatment for OA. However, cell-based therapies derived from adipose tissue are promising. A PRISMA systematic review was conducted employing four databases (MEDLINE, EMBASE, Cochrane, Web of Science) to identify all clinical studies that utilized adipose tissue derived mesenchymal stem cells (AMSCs) or stromal vascular fraction (SVF) for the treatment of knee OA. Eighteen studies were included, which met the inclusion criteria. Meta-analyses were conducted on fourteen of these studies, which all documented WOMAC scores after the administration of AMSCs. Pooled analysis revealed that cell-based treatments definitively improve WOMAC scores, post treatment. These improvements increased with time. The studies in this meta-analysis have established the safety and efficacy of both AMSC therapy and SVF therapy for knee OA in old adults and show that they reduce pain and improve knee function in symptomatic knee OA suggesting that they may be effective therapies to improve mobility in an aging population.

## 1. Introduction

### 1.1. The Burden of Osteoarthritis

Osteoarthritis (OA) is a progressive degenerative joint disorder associated with aging. It is a leading cause of disability around the world. In 2019, the Global Burden of Disease Study reported that musculoskeletal disorders account for over 5% of worldwide disability adjusted life years (DALY) [1]. The World Health Organization (WHO) estimate that approximately 10% of all men and 18% of all women aged over 60 have OA [2]. Out of these individuals, they estimate that 80% have limitations in movement and 25% cannot perform major daily activities of life [2].

In addition to physical symptoms, there is evidence to suggest that OA is associated with mental health problems as well. A longitudinal cohort study, conducted by the Osteoarthritis Initiative, found that there was a greater risk of developing depressive symptoms in patients with hip or knee OA than those without [3]. Another observational study found that OA was associated with 1.27 times increase odds of suicidal ideation [4]. There is also evidence that OA increases the risk for myocardial infarction, with one meta-analysis reporting a 1.31 times increased risk for myocardial infarction [5].

These, among many other studies have highlighted the burden OA has on the individual. In addition to this, OA carries significant economic burden on societies across the world. When adjusted for age, sufferers are shown to be at high risk of sick leave and disability pension due to knee OA. This was especially the case for those working in health care, childcare and in cleaning [6]. In the United States, annual healthcare costs from OA exceed $45 billion [7]. In France, annual costs from OA can be as high as €2 billion [8]. In Spain costs may be as high as €4.738 billion annually [9]. A significant proportion of these costs are associated with joint replacement surgeries.

Economic, societal, and individual burdens caused by OA are set to rise. The prevalence of OA is increasing because of an aging population and an increased incidence of obesity. The United Nations estimates that by 2050, 1 in 6 people in the world will be over 65 years of age [10]. As such incidence rates of OA will naturally also increase. Several projection studies have been performed in different countries. In Australia, the number of people with OA is estimated to increase from 2.2 million in 2015, to 3.1 million by 2030 [11]. In Sweden it is estimated that from between 2012 and 2032, the percentage of people aged over 45 with OA will rise from 26.6% to 29.5% [12]. In the United States, this number is set to grow from 47.8 million in 2005 to 67 million by 2030 [13]. These figures show there is a global rising prevalence of this disease. Coupled with the debilitating nature of OA, this must be addressed before the disease overburdens healthcare systems worldwide.

There is currently no cure to prevent or slow the progression of OA. Presently, it is first managed via conservative means through exercises, weight loss and occupational therapy. When this is inadequate, paracetamol and non-steroidal anti-inflammatory drugs (NSAIDs) are used for symptom control [14]. Intra articular corticosteroid injections are then used if the aforementioned therapies do not provide relief. For end-stage OA, joint replacement surgery (total knee arthroplasty) is the gold standard of treatment. Despite being a highly successful operation, joint replacement surgery carries significant risk, and nearly one in five knee replacements will not last beyond 25 years [15].

OA pathogenesis is predominantly driven by inflammatory mediators such as interleukin 1 (IL-1) and tumour necrosis factor alpha (TNF-α) [16]. Both are present in the synovial fluid of patients with OA [17,18]. IL-1 has been shown to encourage the production of molecules such as nitric oxide, cytokines and prostaglandin E2 [19]. Furthermore, these inflammatory molecules promote the release of matrix metalloproteinases. These encourage the catabolism of articular cartilage [20]. This process of cartilage catabolism and loss is associated with aging, [21] alongside the diminishing ability of cartilage to repair itself [22]. While these mechanisms are central to cartilage depletion and eventually OA and pain, blockade of these mediators have failed to demonstrate efficacy in clinical trials [23,24].

### 1.2. Mesenchymal Stem Cells

MSCs are becoming increasingly popular in tissue engineering due to their multipotent potential to differentiate into different lineages of mesenchymal tissue types [25]. These include bone, fat, cartilage, tendon, and muscle. MSCs are of interest as an OA therapy due to their immunoregulatory function and potential to repair cartilage. This is particularly useful as OA predominantly affects individuals in old age, who have limited ability to repair cartilage.

MSC-based therapies can result in promotion of macrophage polarization from an M1 to M2 phenotype [20,26,27]. This enables macrophages in the cartilage to display anti-inflammatory properties which leads to down-regulation of the inflammatory milieu mentioned above in their role in triggering and sustaining osteoarthritic changes [28]. MSCs have also been shown to be capable of suppressing T-cell proliferation. MSCs do not express HLA class II on their surface and only express low levels of HLA class I and have demonstrated safety and low immunogenicity through various routes of administration [29,30,31]. Although laboratory studies have suggested that MSCs, both intrinsic and transplanted may promote cancer cell activity, in-human clinical trials of transplantation have yet to show evidence of carcinogenic effect [32,33].

The Mesenchymal and Tissue Stem Cell Committee of the International Society for Cellular Therapy recommends a minimal criterion to define human MSCs. There are three elements to these standards. Firstly, MSCs must be adherent to plastic when they are maintained in standard culture conditions. Secondly, MSCs must express the following markers: CD105, CD73 and CD90. In addition, they must not express the following: CD45, CD34, CD14 or CD11b, CD79α or CD19 and HLA-DR surface molecules. Thirdly MSCs must be able to differentiate into the following cell types in vitro: osteoblasts, adipocytes and chondroblasts [34].

MSCs can be derived from a variety of locations including skeletal muscle, synovium [35,36] and periosteum [37,38]. However, the most popular cell sources for MSC harvest, are bone marrow [39,40,41,42] and adipose tissue [43,44]. MSCs were first isolated from bone marrow before any other source [45,46] and bone marrow derived MSCs remain one of the top choices for MSCs due to their high cell-yield and proliferative capacity in vitro [47]. However, despite its advantages, extraction of bone marrow to acquire MSCs for autologous use is a highly invasive and painful procedure that can cause long term pain at the donor site. Thus, such a procedure is not always ideal. Hence, other sources of MSCs have been sought out, the most popular being adipose tissue derived MSCs. These are accessible as a surgical waste tissue and are associated with lower donor site morbidity than bone marrow [48]. The low rejection rates coupled with the anti-inflammatory properties of MSCs makes them an appealing therapeutic solution for OA.

### 1.3. Adipose Tissue Derived MSCs

The most common harvest location for Adipose tissue derived MSCs (AMSCs) is the abdomen due to the high tissue fat content. The harvesting process by which AMSCs are collected have been described extensively in the past [44,45,46,47,48,49,50].

AMSCs have a greater regenerative profile than bone marrow derived MSCs [51]. AMSCs were also found to promote greater neovascularisation, display greater resistance to hypoxia induced apoptosis and higher telomerase activity [52]. Unlike bone marrow derived MSCs, which lose differentiation capacity with age, AMSCs do not [53]. AMSCs maintain their chondrogenic potential and their expansion properties [54]. This is very important to consider in MSC therapies targeted for OA, since these are directed at older patients.

AMSCs have been found to have greater anti-inflammatory properties compared to bone marrow derived MSCs and produce much higher levels of IL-1 receptor antagonist and tissue protective protein tumour-necrosis factor stimulated gene-6 (TSG-6) [55]. When assessed in their role for OA, AMSCs were able to adapt the environment and exerted anti-inflammatory effects on chondrocytes and synoviocytes via prostaglandin E2 [56]. The AMSCs caused polarization of Mo non-polarized macrophages and mature dendritic cells, towards anti-inflammatory and phagocytic phenotypes [57].

Adipose tissue derived stem cells can also be derived from the stromal vascular fraction (SVF) which has the advantage of greater ease of harvest. However, these cells are not plated to select for cells which are plastic adherent [58,59]. As such, cells from the SVF cannot strictly be considered MSCs according to the Mesenchymal and Tissue Stem Cell Committee of the International Society for Cellular Therapy criteria [34].

The aim of this systematic review and meta-analysis was to determine the effectiveness of AMSCs and SVF for the use of treatment in osteoarthritis.

## 2. Materials and Methods

### 2.1. Database and Inclusion Criteria

A systematic review was conducted, based on the guidelines outlined in the Preferred Reporting Items for Systematic Reviews and Meta-Analyses (PRISMA) checklist [60]. Using the PICOS model, (patient, intervention, control, outcome, study), inclusion and exclusion criteria were created. 

A comprehensive literature search was conducted using four databases: Medline (1946 to 4 June 2020), EMBASE (1974 to 29 June 2020), Cochrane library (1946 to June 2020), and Web of Science (1900 to 2020). 

The following was used as inclusion criteria for all studies screened:

1. Any studies which investigated use of AMSCs on humans for the treatment of knee joint osteoarthritis
2. Any study that included the use of stromal vascular fraction (SVF) or microfragmented adipose tissue
3. Any study which was clinical in nature

Consequently, the following was used as exclusion criteria for all studies screened.

1. Any study not conducted on human
2. Studies which investigated use of MSCs which were not of adipose origin
3. Any case studies and reviews
4. Studies in which the data sets were either incomplete or inaccessible such as conference abstracts and ongoing randomised controlled trials (RCTs)

**Figure 1:**
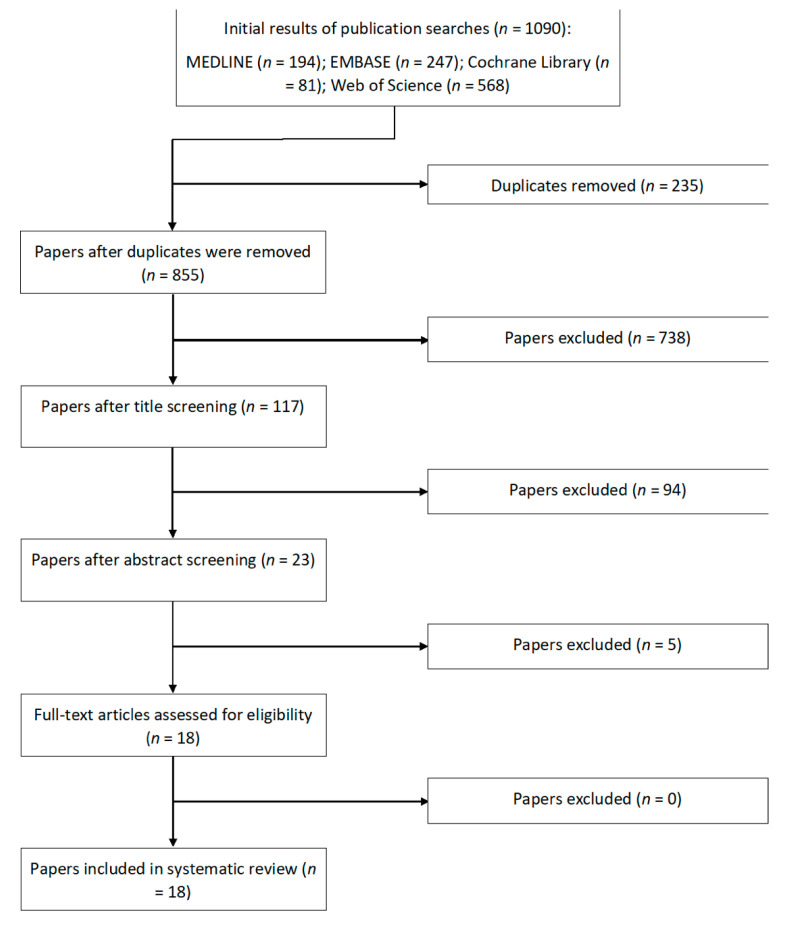
Overview of the screening and selection process of studies for the systematic review.

A search strategy was created, on the basis of the Cochrane Highly Sensitive Search Strategy. This included but was not limited to the following terms: ‘mesenchymal’, or ‘stem cell’ and ‘osteoarthritis, knee’ and ‘adipose’. Full search strings can be found in the Appendix A. Restrictions were applied to the search to only include studies conducted on humans and in the English language. Study selection was carried out by two reviewers independently. The titles of the articles were reviewed for relevance. Abstracts were then screened to check if they met inclusion criteria. Full-text manuscripts were then retrieved and analysed. A manual search was also performed on associated review articles to identify any articles that could have been missed by the search. The combined results of the comprehensive search strategy are shown in Figure 1. 

### 2.2. Quality Assessment

Each study was critically appraised to ensure relevance. Studies were appraised by two independent reviewers using either the Risk of Bias in Nonrandomised Studies 1 (ROBINS-1) or the Risk of Bias 2 (RoB2) for randomised studies tools. Upon completion of the appraisal, data was stratified according to the tools used and was collated into tables (Supplementary Tables S1 and S2). Any uncertainty was solved through discussion between the reviewers. 

### 2.3. Statistical Analysis

Pre and post treatment Western Ontario and McMaster Universities Osteoarthritis Index (WOMAC) scores were extracted from each study for each of the follow-up times. Percentage changes in these scores were then calculated for each follow-up time. Forest plots were created for each of the follow-up time periods. A summary forest plot was created to determine the overall statistical significance of treatment on WOMAC scores. We sought to elucidate whether the pooled effect of treatment resulted in minimum clinically important differences in WOMAC scores, which has been defined as 12, [61] to this end, we do not compare this against the various control groups used across the studies. All statistical analysis was conducted on R software through the ‘metafor’ package. The I^2^ test was used to test for heterogeneity. To account for heterogeneity (all-cause) between studies, assuming effects assuming that effects between studies are either similar or not, Fixed effect models were used for analyses with I^2^ < 25%. Random effect models were used for analyses with I^2^ > 25%. All WOMAC percentage changes were calculated with a 95% confidence interval. 

## 3. Results

The search conducted on Medline (1946 to 4 June 2020), EMBASE (1974 to 29 June 2020), Cochrane library (1946 to June 2020), and Web of Science (1900 to 2020) included 117 potentially relevant articles after title screening, and 94 studies were excluded after abstract screening as these were non-human, preclinical studies or did not assess the treatment of interest. After full textual analysis five studies were removed as these did not include quantitative treatment outcomes relevant to our study, only eighteen studies were then included in this review. One of the 18 studies was a two-year follow-up of another included study [62]. The original study was a prospective cohort study which investigated the effects of three different AMSC dose injections [63]. Out of the studies, 16 were prospective [43,49,62,63,64,65,66,67,68,69,70,71,72,73] and two were retrospective [74,75]. Only five were randomised controlled trials [48,49,64,69,70,71,72]. The general characteristics of each AMSC study can be seen in Table 1. All studies investigated autologous treatments. Characteristics for SVF studies can be found in Table 2. Cellular characteristics for AMSC studies and SVF studies can be found in Table 3 and Table 4, respectively. Outcomes measured, and complications documented have been collated for each study in Table 5. The overall bias determined in all studies included in this systematic review were found to be low, indicating high quality studies.

**Figure 2:**
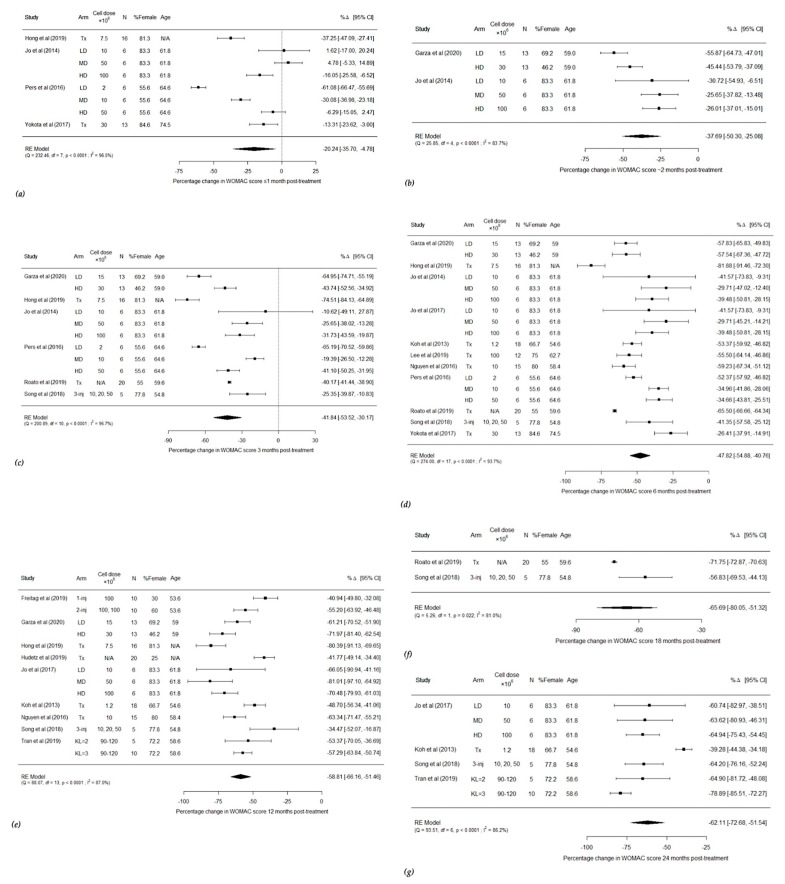
Forest plots showing percentage changes in WOMAC score less than one month after treatment (**a**), approximately two months after treatment (**b**), three months after treatment (**c**), six months after treatment (**d**), twelve months after treatment (**e**), eighteen months after treatment (**f**) and twenty-four months after treatment (**g**) (1-inj = 1 injection, 2-inj = 2 injections, 3-inj = 3 injections, CI = confidence intervals, df = degrees of freedom, HD = high dose, LD = low dose, MD = middle dose, N = number, RE = random effects, Tx = treatment group).

Forest plots were created, according to the follow-up time periods utilised, to analyse changes in WOMAC scores post AMSC and SVF treatment (Figure 2) in the treatment group.

Figure 2a shows all four studies which documented WOMAC scores less than 1 month after treatment [63,68,70,72,78]. There was a statistically significant improvement in WOMAC scores in the individual arm studies, and in the high dose groups of the other studies. The pooled analysis showed a −20.24% change [95% CI −35.70, −4.78] suggesting that use of AMSCs and SVF resulted in a statistically significant improvement in WOMAC scores less than a month after treatment [Q = 232.46, *p* < 0.0001]. Figure 2b–g show all studies which evaluated WOMAC scores at approximately two months, three months, six months, twelve months, eighteen months and twenty-four months after treatment, respectively [48,49,62,63,64,66,67,68,69,70,71,72,73,78] The plots show that there are statistically significant improvements in WOMAC scores in all arms of all treatment groups across the follow-up periods between two and twenty-four months. The pooled analysis was −37.69% change [95% CI −50.30, −25.08, Q = 25.85, *p* < 0.0001] post two month treatment, −41.84% [95% CI −53.52, −30.17, Q = 200.89, *p* < 0.0001] post three month treatment, −40.07% [95% CI −54.44, −39.65, Q = 273.47, *p* < 0.0001] post six month treatment, −58.44% [95% CI −66.41, −50.47, Q = 85.71, *p* < 0.0001] post twelve month treatment, −65.69% [−80.05, −51.32, Q = 5.26, *p* = 0.022] post eighteen month treatment and −62.11% [95% CI −72.68, −51.54, Q = 93.51, *p* < 0.0001] post twenty-four month treatment. 

**Figure 3:**
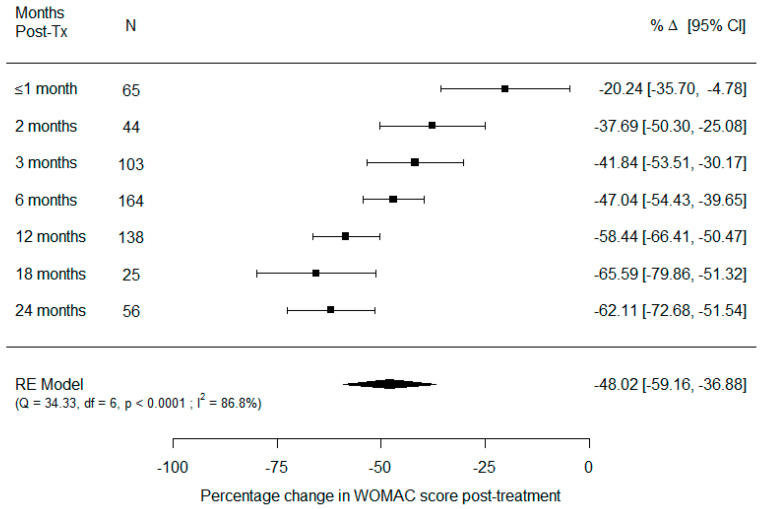
Forest plot showing pooled analyses of percentage changes in WOMAC scores across the different follow-up times (CI = confidence intervals, df = degrees of freedom, N = number, RE = random effects, Tx = treatment group).

A forest plot was created to show a summary of the pooled analyses for the respective follow-up times (Figure 3).

Figure 3 shows the pooled analyses for all the follow-up time periods. In every follow-up, post-treatment, there was an improvement in WOMAC scores. This improvement increased as time went on, between less than month and up to 18 months post-treatment. There was a slight reduction in improvement at 24 months, compared to 18 months. These analyses were further pooled showing −48.02% [−59.16, −36.88, Q = 34.33, *p* < 0.0001]. This suggests that overall, there was a statistically significant improvement in WOMAC scores post treatment.

### 3.1. Classification of Osteoarthritis

Most studies in the literature used the Kellgren-Lawrence (KL) radiological classification of osteoarthritis to grade the severity of OA in patients [48,49,62,63,64,66,67,68,69,70,71,73,74,75,78]. Due to the subjective nature of clinical diagnoses of OA, this was not used in any studies. Several of the included studies used this classification in their inclusion and exclusion criteria where for example, patients with a KL grade of one would not be included [48,49,63,64,66,67,68,69,71,72,75,78] in the study. Some studies also only included patients who had an average pain intensity of four or more on the 10-point visual analogue scale for at least four months [48,63,64,70]. One study used the IKDC classification to grade OA [65] and another used the Brandt Radiographic Grading Scale for Osteoarthritis [77].

### 3.2. Follow-Up

The most common follow-up period was 6.5 months [63,64,72,74]. Three studies had a follow-up of 12 months [69,70,71]. Three studies had a follow-up of 24 months [38,60,61]. One study had a follow-up of one month [68]. Five studies had a follow-up between 12 and 24 months [49,65,67,73,74]. Two studies had a follow-up greater than 24 months [62,66]. 

### 3.3. Adverse Events 

Only two studies have been found which did not report whether adverse events (AEs) or severe adverse events (SAEs) occurred during the clinical study [65,78] The rest of the studies all reported AEs. Two studies observed no AEs or SAEs during the study [67,71]. One of these studies however had four cases of complications which were deemed unrelated to the treatment regimen. These complications were high blood pressure, chest pain, dyspnoea, and urinary retention [67]. Eleven studies reported that subjects commonly experienced either transient pain or swelling of the joint after injection of the AMSCs or SVF [48,49,64,65,66,67,68,69,70,72,73,74,77]. In most patients this resolved spontaneously. In some patients, paracetamol was administered after which this resolved. Some studies also reported that subjects experienced discomfort at the site of lipoharvest [49,68,74]. However, this was resolved on further follow-up. Three studies reported patients experienced internal haematomas at the site of lipoharvest [49,74]. Four studies reported SAEs [49,63,64,65,66,72]. One subject in one study had a urinary stone. The subject had a past medical history of stones, and this was subsequently treated [63]. In another study, a subject experienced angina pectoris. However, they had risk factors of hypertension and hyperlipidaemia which predisposed them to the condition. In the remaining two studies, two patients and one patient respectively experienced severe pain and swelling following the procedure [49,66]. The two patients recovered after four weeks, while the single patient recovered after two weeks. 

### 3.4. Outcome Measures 

Several studies recorded two primary outcomes: clinical and radiological outcomes. Studies utilised patient reported outcome measures (PROMs) to document clinical outcomes and the PROMs used varied greatly between studies (Table 4 and Table 5). The most widely employed scoring systems were the Western Ontario and McMaster Universities Osteoarthritis Index (WOMAC), and the visual analogue scale (VAS). The former was used by fifteen studies, while the latter was used by fourteen studies. The Knee Injury and Osteoarthritis Outcome Score (KOOS) was used by six studies. The Knee Society Score (KSS) was used by three studies. The Lysholm score was also employed by three studies. The Numeric Pain Rating Scale (NPRS), range of motion (ROM) and bone marrow oedema (BME) scoring were utilized by two studies. The Short Form-36 (SF-36), Hospital for Special Surgery Knee score (HSS), Patient Global Assessment score (PGA), Short Arthritis Assessment scale (SAS), International Knee Society (IKS) score, Japanese Knee Osteoarthritis Measure (JKOM), Joint Motion Amplitude (JMA) and the six minute walking distance score (6MWD) were each utilized by one study.

Fewer papers assessed radiological outcomes. Six studies used radiographs to assess KL grades of patients after the said treatment was given. Several studies utilised Magnetic Resonance Imaging (MRI) to assess cartilage defects in the knee of patients. However, only seven of these used standardised radiological scoring systems. Freitag et al., (2019) used the MRI Osteoarthritis Knee Score (MOAKs). Koh et al., (2013) utilised the Whole-Organ MRI Score (WORMS) [66]. Spasovski et al. (2018) used the 2D Magnetic Resonance Observation of Cartilage Repair Tissue (MOCART) score [65]. Hong et al., (2019) used both WORMS and MOCART [70]. Nguyen et al., (2016), Garza et al., (2020) and Tran et al., (2019), all used the Outerbridge Classification System (OS) [67,69,78].

Only two studies conducted a second look arthroscopy and subsequent histological analysis of the cartilage. Jo et al., (2014) used the International Cartilage Repair Society (ICRS) score for histological grading [63], while Pers et al. (2016) used Osteoarthritis Research Society International (OARSI) for histological grading [72].

## 4. Discussion

End-stage knee OA is currently managed with joint replacement surgery. This, however, does not target the underlying disease process of OA, but rather the end stage symptoms. Treatment options such as cartilage repair and osteotomy can delay the progression of OA, but do not modify the disease [79,80]. Recently, use of AMSCs has sprung into the clinical purview. AMSCs have the potential to regenerate new healthy articular cartilage and thus alleviate the symptoms of knee OA. The results of this systematic review and meta-analysis demonstrate that use of both AMSC and SVF treatments significantly reduce WOMAC pain scores. This suggests that these treatments provide improved function and a reduction in pain.

Numerous advantages of AMSCs have been described in the literature [76,81]. However, despite this, there is limited information on this topic in the literature, especially with regards to human studies. Animal studies are more widespread since the safety of use of AMSCs had to first be established, this was first conducted in mice. ter Huurne et al. (2012) conducted such a study (C57BL/6 mice), with early-stage collagenase induced OA. They found that injection of AMSCs into the knee joints of these mice, led to a reduction and inhibition of cartilage destruction and formation of enthesophytes. In addition, there was reduced synovial thickening and the treatments were safe.

Use of mice studies, have allowed the evaluation of safety of AMSC transplantation to treat knee OA. Song et al., (2018) conducted a human clinical trial using adipose tissue derived stem cells. However, they first conducted preclinical safety tests in vitro and on BALB/c-nu nude mice. After confirming the safety of administering AMSCs isolated through their methodologies, they enrolled 18 patients in their clinical study. These patients were separated into three groups: low-dose (1 × 107), mid-dose (2 × 107), and high-dose (3 × 107). Each patient was injected three times and followed up over a course of 96 weeks. They found that use of AMSCs is safe for human use.

This study and several others documented AEs and SAEs. However, despite these occurrences, they did not cause long term detriments to the patients’ quality of life and in most cases spontaneously resolved. As such several studies deemed AMSC therapy to be safe [48,49,63,65,72,73,74].

AMSC or SVF post treatment outcomes were determined by changes in WOMAC scores in fourteen of the eighteen studies included in this systematic review. One study conducted by Bansal et al. (2017) also used WOMAC scores, however since standard deviation or error figures were not provided, this was excluded from quantitative meta-analysis [77].

In the main pooled analysis, it was demonstrated that use of AMSCs and SVF in knee osteoarthritic joints improved WOMAC scores. All studies that documented WOMAC scores between two months and 24 months after treatment found that there was a statistically significant improvement in WOMAC scores after treatment. This was the case, regardless of the dose of AMSC or SVF used, or number of injections administered, suggesting that the less laborious preparation of SVF compared to MSCs may be an advantage as both therapies achieve good clinical outcomes. Furthermore, the pooled analyses in Figure 3 illustrates that there is a statistically significant improvement in WOMAC scores across all follow-up times, suggesting disease modification that persists and long-term efficacy without the need for repeat administration of treatment. These improvements increased as time from initial treatment increased between less than one month and eighteen months post treatment. After this, at twenty-four months there was a slight decrease in improvements in WOMAC scores. Overall, this suggests that the therapies act beyond short-term analgesia, and lead to changes in the disease process. Improvements in these scores suggest that these AMSC and SVF treatments reduce pain and improve knee function in patients with knee OA. Due to the low number of studies that compared AMSC and SVF therapies, we were unable to conduct a meta-analysis to directly compare these therapies. As there were also significant heterogeneity in the studies, subgroup analyses of AMSC and SVF separately could not be conducted to yield meaningful results. The pooled analysis in this review offers findings that are generalizable to multiple adipose derived cell-based therapies.

Out of the eighteen studies included in this review, five investigated the effects dose of AMSCs, had on outcomes [48,62,63,72]. In four of the studies, patients were divided into three groups: a low dose group, medium dose group and high dose group. Three of these studies found that patients in the high dose group gained greater clinical improvements than those in the low and medium dose groups. This suggests that there is a relationship between the number of AMSCs administered and the therapeutic effect gained. This seems intuitive, as joint-native MSCs in OA patients may have diminished capacity to proliferate and repair cartilage [82]. Thus, provision of healthy, functional MSCs could prevent further cartilage loss and repair existing defects. This is in line with conclusions made by two studies which investigated radiological outcomes. They discovered greatest reduction in cartilage defects were found in the high dose groups [62,63]. One can posit that this suggests that increasing dose is correlated with greater regenerative potential. Conclusions drawn by Pers et al., (2016) differed with the aforementioned studies, finding that the lowest dose group had the best improvements in clinical outcomes. This may have arisen, since the authors documented that the patients with the highest levels of inflammation were those in the low dose group [72]. AMSCs could have been primed to exert their immunoregulatory functions more efficiently in this more prominent inflammatory background. However other studies in the literature show similar findings, implying that lower dose administration of AMSCs may be more effective than high dose [83,84]. If low doses of AMSCs, are just as or more effective than high doses, then clinical and radiological improvements do not correlate with the number of cells used. Using less cells may reduce the laborious preparation required. Moreover, this may cut down on costs, making AMSCs more appealing for clinical use. Since very few studies investigated doses on outcomes, subgroup analyses were not conducted.

Freitag et al., (2019) compared the effect one injection of 100 × 10^6^ cells, to two injections of 100 × 10^6^ cells. Both were compared to a control group. They concluded that two injections of AMSCs achieved more consistent OA stabilisation than one injection [49]. They were the only study to do this, which represents a gap in the literature. This indicates that multiple low dose injections of AMSCs may provide superior clinical and radiological improvements than a singular dose. These results may be reflective of the overall increased number of cells used in the double injection group. On the other hand, this increased efficacy of the double injection treatment could be due to spaced out exposure to AMSCs. Repetitive low dose spaced out injections of AMSCs may prove to be more successful than a single large dose injection. Nevertheless, there is also a possibility that there is a ceiling on the correlation between numbers of injections and improvements seen. Moreover, Freitag et al., (2019) reported an increase in the number of moderate AEs in the second injection group. This implies that increased numbers of injections are linked to increased AEs, which may affect the tolerate of the therapy in question.

Yakota et al. (2019) was the only study that compared the efficacy of AMSCs with SVF [74]. They found that both therapies improved osteoarthritic symptoms and pain. However, when they analysed the clinical scores, they found that the improvements were more significant and occurred earlier in the AMSC group. Earlier clinical improvements suggest that AMSCs have a faster mechanism of action. More significant clinical improvements suggest that AMSCs are superior to SVF for symptomatic control of knee OA. No radiological outcomes were investigated in this study. Therefore, no conclusions can be drawn regarding the cartilage regenerative potential of either treatment. However, other studies have shown that AMSCs have great cartilage regenerative potentials, and reduce cartilage defects [62,63]. Yakota et al., (2019) also discovered that there was a higher frequency of knee effusion and minor complications related to the harvesting of adipose tissue in the SVF patient group [74]. As such iatrogenic complications may be higher in SVF. This may affect tolerate and favourability of such treatment in the future. More clinical studies need to be conducted to make robust conclusions regarding which treatment is superior.

Garza et al., (2020) was the only study to compare the effects of different doses of SVF on clinical and radiological outcomes. They led a study in which they compared a placebo group using hyaluronic acid, with a low dose SVF group (1.5 × 107 cells) and a high dose SVF group (3.0 × 107 cells) [69]. They discovered both doses of SVF resulted in improved WOMAC scores compared to the placebo. Nevertheless, there was no statistical difference between the high and low dose groups. Therefore, this suggests that the benefits expressed by SVF is not dose dependent. Additionally, there was no visible quantifiable changes detected in cartilage thickness on the MRI scans between all three groups. The result from the radiological outcomes suggests that unlike AMSCs, which improve cartilage defects, SVF do not. Therefore, SVF treatment has no impact on the disease process of OA and only plays a role in symptomatic relief. This could be potentially explained by the fact that AMSCs have a higher number of colony forming fibroblast units (CFU-F) and greater differentiation potential [58]. This study also further illustrates the limitations of hyaluronic acid for the treatment of knee OA. This review has found a gap in the literature, which must be addressed with studies investigated SVF dose and outcomes, to further determine the role of SVF therapy in the treatment of knee OA.

Koh et al., (2013), Bansal et al. (2017) and Nyugen et al. (2016) were the only studies which used a growth factor alongside SVF [66,67,77]. They all used a platelet rich plasma (PRP) scaffold. All studies concluded that there was significant improvement in clinical scores long term post treatment. Use of PRP may have improved the efficacy of treatments. PRP is known to enhance MSC proliferation and chondrocyte differentiation, and as such could bolster cartilage degeneration [85,86]. However, none of these studies compared use of PRP alone against SVF and PRP. As such the efficacy of PRP cannot be quantified. Only with further studies, comparing AMSCs and SVF with and without PRP can we definitively determine the role of PRP in therapy for knee OA. If use of growth factor proves to increase efficacy, these could be applied to AMSC therapy as well.

Many of the studies included in this systematic review used K-L grading of OA as an inclusion and exclusion criterion in the recruitment of their patients. Out of all studies, only one included patients with a K-L grade of 1 [73]. As such most of the studies could not determine the efficacy of treatments on low grade knee OA. Since, there is reduced levels of inflammation in the earlier stages of OA than end stages, AMSC and SVF therapy may be more effective. Alternatively, these therapies may be more effective in end stage OA, since the high inflammatory environment could modulate cells to exercise their immunoregulatory function more effectively. In addition, only eight studies included patients with K-L grade 4 [48,62,63,64,68,71,72,74]. As such there is less data on the effects of AMSC treatment on end stage severe knee OA, and more on middle stage knee OA. Furthermore, most studies did not stratify patients according to the severity of the OA. As such they could not determine whether the severity of knee OA has any bearing on the efficacy of the MSC treatment. Nonetheless, three of the studies did make observations based on this. Tran et al., (2019) inferred that treatment was more effective in patients with KL grade 3 than with grade 2 [78]. In the higher severities of OA, there is greater inflammation. On the other hand, Nyugen et al., (2016) and Yokota et al., (2019) found that lower K-L grades had greater clinical improvements, indicating that efficacy was greater in patients with less severe OA [67,74]. If this is the case, more studies need to be conducted to include K-L grade 1 patients.

In addition to K-L grading, studies did not stratify patient cohorts according to age or BMI. Thus, the impact these could have on the efficacy of outcomes is unknown. It is possible that in younger patients, who have superior regenerative potential, the quality and therefore efficacy of AMSC and SVF is superior to elderly patients [87]. Maredziak et al. (2016) illustrated that there is reduced CFU-F, proliferation rates, and quantified chondrogenic and osteogenic differentiation in aged AMSC cells. Furthermore, aged cells seem to shift more in favour of adipogenic differentiation [88]. In addition, it has been shown that there is a biological role of adipose inflammation in obese patients and OA [89]. As such, it is possible, this mechanism of OA may respond differently to AMSC and SVF treatments, compared to age related articular cartilage degeneration. Therefore, stratifying patients into different BMI groups, may be of benefit. However, this must be investigated further before definitive conclusions can be made.

The gold standard of evaluating new-born cartilage in the face of cartilage repair, is second look arthroscopy and histological biopsy. It is important to determine quantify the size of cartilage regeneration as well as the constitution of the cartilage. Nevertheless, only two studies performed such procedures [63,72]. To confidently determine the role of AMSC treatment in knee OA, we must understand the qualities and mechanism of cartilage repair involved.

Out of all the studies, six used arthroscopies prior to injection of the treatment. Roata et al. (2019) and Jo et al., (2017) only used arthroscopy as guidance for injection of the AMSCs into the osteoarthritic site [62,73]. However, the rest of the studies performed arthroscopic debridement prior to injection of the treatment [67,70,74]. As such it is possible that this led to bias of outcome. Due to the heterogeneity in treatment modality between the studies, and the low number of studies that examined arthroscopic delivery, sub-group analysis was not conducted. It should be noted that arthroscopy for the treatment of knee OA has been shown to be largely ineffective [90,91]. However, arthroscopic debridement removes inflammatory synovial fluid which can interfere with AMSC adhesion in vitro, and therefore increasing the effectiveness of said treatment [73]. As such, arthroscopic debridement may prime the joint to become more responsive to injections. Consequently, one cannot definitively rule out the effect arthroscopy has on the clinical and radiological outcomes of AMSC treatment.

Five of the studies included, utilised ultrasound to aid guidance of the AMSC or SVF injections. When the outcomes of these studies are compared to those which used arthroscopy or utilised neither, there is no difference in clinical outcomes. As mentioned previously, only two studies conducted second look arthroscopies. Hence, we cannot determine whether use of image guidance leads to greater cartilage regeneration. Future studies should directly compare use of image guidance against blind injection. In addition, studies should perform radiological and histological analysis on all patients to determine if imaging guidance has any bearing on cartilage regeneration.

There was a lack of long-term studies carried out, as shown in the results. The average follow-up across all studies was 60.1 weeks. The longest follow-up period was 104 weeks [62]. This would be considered a short-term follow-up. Thus, there is a gap in the literature for such long-term studies. As a result, the effect AMSC injections and SVF injections have on long term knee OA is unknown. The importance of this was made evident by Park et al., (2016). They conducted a study in which they investigated cartilage regeneration in OA patients, through use of umbilical cord derived MSCs. They discovered that three years after treatment, cartilage repairs persisted. This implied that use of such MSCs could provide a long-term solution to OA. Conversely, Jo et al., (2017) found that two years post treatment, cartilage deterioration was apparent. As such, it is possible that the effect of AMSC and SVF treatment may be limiting, and further injections may be required for persistence of cartilage regeneration. Alternatively, it could be possible after several years, the knee joint becomes unresponsive to AMSC and SVF treatment injections. Only with long term studies, can the lasting implications of this treatment be determined. Understandably, the studies included in this review are very recent, as such long-term studies with 5–10-year follow-ups are not possible at this stage.

There are significant variations in the outcome measures utilised by studies. In terms of clinical outcomes, most studies using WOMAC and VAS. However, several did not. As a result, the clinical outcomes recorded are harder to compare between studies. Scoring systems such as WOMAC and VAS are non-specific. Perhaps creation of a PROM specifically for post intra-articular injection would be more beneficial. Use of a handful of scoring systems rather than a wide array may allow for better comparison between studies and therefore allow scientists to determine what the most effective treatment for knee OA.

SVF can be prepared through various methods. It has been shown that different methods have different compositions and properties [92]. This has not been addressed by any of the studies included in this review which investigated SVF. As a result, it may be unwise and inaccurate to compare the results of studies which used differing methods to produce their SVF.

The gold standard in medical research when testing the efficacy of a new treatment is a double blinded randomised controlled trial (RCT). However, use of AMSCs is very novel. Many of the studies included in this systematic review were carried out as pilot studies. Out of these studies, only six used a control group [49,64,67,69,70,78] As such the other studies are unable to truly define the efficacy of the treatments tested. Many of these studies recognised this, however their principal goal was to determine the safety of AMSCs rather than its effectiveness. They stated that future studies should include control arms in clinical trials. However, Freitag et al., (2019) and Pers et al., (2016) believed there may be ethical concerns with conducting an RCT [48,49,50,51,52,53,54,55,56,57,58,59,60,61,62,63,64,65,66,67,68,69,70,71,72,73,74,75,76,77,78]. All patients would have to undergo a lipoharvest procedure before randomisation. This procedure is not without complications. A study conducted by Comella et al. (2017) found that such complications are low in lipoharvest procedures [93]. Since these studies were pilot in nature, their sample sizes were also very small, with the largest being 80 patients, and the average being 30.5 across all studies.

## 5. Conclusions

The studies in this systematic review have established the safety and efficacy of both AMSC therapy and SVF therapy for knee OA in humans. In addition, the meta-analyses show that use of AMSC and SVF therapy for knee OA definitively improves WOMAC scores up to two-years, with improvements increasing with time. This suggest that AMSC and SVF treatments reduce pain and improve knee function in patients with severe knee OA. Future clinical studies must now incorporate control arms and have larger sample sizes to successfully determine the effectiveness of these treatments. Specifically, there is little to no literature on studies comparing use of single vs multiple AMSC injections, comparing AMSC treatments with SVF treatments and the effect of different doses of SVF on outcomes.

## Figures and Tables

**Table 1:** Study characteristics for AMSC studies, *n* = 11.

Authors	Type of Study	Treatment vs. Control	No. of Patients in Control Group (Gender)	No. of Patients in Treatment Group (Gender)	Age, Mean	Location of Defect	Grade of OA (Grading Classification)
Lee et al. (2019) [64]	Prospective double blinded RCT	AMSC at 1 dose, control	12 (3M, 9F)	12 (3M, 9F)	62.7	Knee	II–IV (K-L)
Freitag et al. (2019) [76]	Prospective non blinded RCT	AMSC at 2 doses, control	10 (5M, 5F)	10 (7M, 3F) for 1 injection. 10 (4M, 6F) for 2 injections	53.6	Knee	II–III (K-L)
Song et al. (2018) [48]	Prospective double blinded RCT	AMSC at 3 doses, no control	N/A	18 (4M, 14F)	54.8	Knee	II–III (K-L)
Roato et al. (2019) [73]	Prospective single arm study	AMSC at 1 dose, no control	N/A	20 (9M, 11F)	59.6	Knee	I–III (K-L)
Hudetz et al. (2019) [63]	Prospective non-randomised trial	AMSC injection, no control	N/A	20 (15M, 5F)	Not specified	Knee	III–IV (K-L)
Spasovski et al. (2018) [65]	Prospective single arm study	AMSC at 1 dose, no control	N/A	9 (3M, 6F)	63	Knee	B-D (IKDC)
Jo et al. (2017) [62]	Prospective cohort study	AMSC at 3 doses, no control	N/A	18 (3M, 15F)	61.8	Knee	III–IV (K-L)
Bansal et al. (2017) [77]	Prospective interventional	AMSC injection, no control	N/A	10 (6M, 4F)	58.4	Knee	I–II (BS)
Pers et al. (2016) [72]	Prospective single arm study	AMSC at 3 doses, no control	N/A	18 (8M, 10F)	64.6	Knee	III–IV (K-L)
Jo et al. (2014) [63]	Prospective cohort study	AMSC at 3 doses, no control	N/A	18 (3M, 15F)	61.8	Knee	III–IV (K-L)
Yokota et al. (2019) [75]	Retrospective cohort study	AMSC vs SVF, no control	N/A	80 (16M, 64F)	71.4	Knee	II–IV (K-L)

AMSC = Adipose Tissue Derived Mesenchymal Stem Cell, F = Female, IKDC = International Knee Documentation Committee, K-L = Kellgren-Lawrence, M= Male, N/A = Not Applicable, OA = Osteoarthritis, RCT = Randomised Controlled Trial.

**Table 2:** Study characteristics for SVF studies, *n* = 7.

Authors	Type of Study	Treatment vs. Control	No. of Patients in Control Group (Gender)	No. of Patients in Treatment Group (Gender)	Age, Mean	Location of Defect	Grade of OA (Grading Classification)
Garza et al. (2020) [69]	Prospective double blinded RCT	High dose SVF vs low dose SVF vs placebo	13 (6M, 7F)	13 (4M, 9F) for low dose SVF. 13 (7M, 6F) for high dose SVF.	59.0	Knee	II–III (K-L)
Hong et al. (2019) [70]	Prospective RCT	One knee with SVF and the other with hyaluronic acid placebo	16 (3M, 13F)	16 (3M, 13F)	Not specified	Knee	II-III (K-L)
Tran et al. (2019) [78]	Prospective non-randomised trial	Arthroscopic microfracture vs arthroscopic microfracture and injection of SVF	15 (3M, 12F)	18 (5M, 13F)	58.64	Knee	II–III (K-L)
Yokota et al. (2017) [68]	Prospective uncontrolled	Injection of SVF, no control	N/A	13 (2M, 11F)	74.5	Knee	III–IV (K-L)
Nguyen et al. (2016) [67]	Prospective unblinded, non-randomised trial	Arthroscopic fracture vs arthroscopic fracture and injection of SVF and PRP.	15 (3M, 12F)	15 (3M, 12F)	58.4	Knee	II–III (K-L)
Koh et al. (2013) [66]	Prospective cohort study	SVF at 1 dose with PRP, no control	N/A	18 (6M, 12F)	54.6	Knee	III–IV (K-L)
Panni et al. (2019) [74]	Retrospective single arm study	SVF at 1 dose following arthroscopy (for chondral shaving/abrasion and/or meniscal regularization), no control	N/A	52 (22M, 30F)	57.3	Knee	0–II (K-L)

BS = Brandt Radiographic Grading Scale for Osteoarthritis, F = Female, K-L = Kellgren-Lawrence, M= Male, N/A = Not Applicable, OA = Osteoarthritis, RCT = Randomised Controlled Trial, SVF = Stromal Vascular Fraction.

**Table 3:** Cellular characteristics for AMSC studies, *n* = 11.

Authors	Number of Cells Used/Multiple Injections	Method of Delivery of Cells	MSC Pre-Treatment	Follow-Up Period (Weeks)	Harvest Site	Method of Harvest	MSC Surface Marker Validation Via Flow Cytometry
Lee et al. (2019) [64]	1 × 10^8^ cells	Intra articular injection under USS guidance into unspecified joint space	Adipose tissues were treated with collagenase I and were centrifuged to obtain a pellet which was resuspended in culture media. The cells were cultured for up to 5 days in media until confluent and were then harvested at passage 3	26	Abdomen	Liposuction	AMSCs were positive for CD73, CD90. AMSCs were negative for CD31, CD34, CD45
Freitag et al. (2019) [49]	100 × 10^6^ cells, single and double injection	Intra articular injection under USS guidance into unspecified location in joint space	Lipoaspirate was digestion followed by centrifugation. MSCs were cultured under hypoxic conditions with standard growth media until 80% confluency and was expanded to passage 2.	52	Abdomen	Liposuction	AMSCs were positive for CD73, CD90, CD105, AMSCs were negative for CD14, CD19, CD34, CD45
Song et al. (2018) [48]	1 × 10^7^, 2 × 10^7^ and 5 × 10^7^ cells, three injections	Intra articular injection under USS guidance into unspecified location in joint space	Lipoaspirated suspensions were digested and centrifuged, then cells were culture-expanded to passage 4.	96	Not specified	Liposuction	AMSCs were positive for CD29, CD49d, CD70, CD90 and were negative for actin, CD13, CD34, CD45, HLA-DR
Roato et al. (2019) [73]	Not specified	Intra articular injection under arthroscopic guidance into chondral defect site	Lipoaspirate was treated with Collagenase. The resulting cell pellet was then resuspended into culture media and counted.	78	Abdomen	Liposuction	AMSCs were positive for CD73, CD90, CD105, IgG1, IgG2a AMSCs were negative for CD44, CD45
Hudetz et al. (2019) [71]	Unspecified	Intra articular injection into unspecified location in joint space	Samples were digested with collagenase and samples were filtered through a 100 μm cell strainer and centrifuged. The cell pellet was resuspended in DMEM.	48	Abdomen	Liposuction	AMSCs were positive for CD70, CD90, CD105, CD146. AMSCs were negative for CD31, CD34, CD45.
Spasovski et al. (2018) [65]	0.5–1 × 10^7^ cells	Intra articular injection into unspecified location in joint space	MSCs were digested using collagenase, expanded in standard culture media and harvested between passage 2 and 4.	78	Abdomen	Liposuction	AMSCs were positive for CD73, CD90, CD105. AMSCs were negative for CD34, CD45
Jo et al. (2017) [62]	1 × 10^7^, 5 × 10^7^ and 1 × 10^8^ cells	Intra articular injection under arthroscopic guidance into unspecified location in joint space	Aspirated tissues were digested with collagenase I. Cells were cultured for 4-5 days until confluent. All AMSCs used in this study were collected at passage 3.	104	Abdomen	Liposuction	AMSCs were positive for CD73, CD90. AMSCs were negative for CD14, CD34, CD45
Bansal et al. (2017) [77]	1 × 10^6^ cells	Intra articular injection into unspecified location in joint space	The adipose tissues was filtered and centrifuged. The cell pellet was re-suspended in culture medium and the media was changed every 3-4 days until the cells achieved 90% confluency.	96	Abdomen	Liposuction	AMSCs were positive for CD70, CD90, CD105. AMSCs were negative for CD34, CD45, HLA-DR
Pers et al. (2016) [72]	2 × 10^6^, 10 × 10^6^ and 50 × 10^6^ cells	Intra articular injection under USS guidance into unspecified location in joint space	Adipose tissue was digested with collagenase solution and plated in culture medium. Cells were passaged and then cultured in CCM for 14 days with media changes every 3–4 days until confluence.	26	Abdomen	Liposuction	AMSCs were positive for CD73, CD90, CD105, IgG1 AMSCs were negative for CD31, CD34, CD45
Jo et al. (2014) [63]	1 × 10^7^, 5 × 10^7^ and 1 × 10^8^ cells	Intra articular injection under arthroscopic guidance into unspecified location in joint space	Aspirated tissues were digested with collagenase and cells were resuspended in media until confluent. AMSCs used were collected at passage 3.	26	Abdomen	Liposuction	AMSCs were positive for CD73, CD90. AMSCs were negative for CD31, CD34, CD45
Nakamura et al. (2019) [75]	12.75 × 10^6^ cells, unknown for SVF	Intra articular injection into unspecified location in joint space	The collected aspirate was digested with collagenase. Cells were cultured in medium that was replaced every 3 days thereafter. When cells reached 80% confluency they were passaged up to four times. SVF cells were produced without culture in a sterile single-use functionally-closed system, requiring approximately 2–2.5 h from lipoaspirate.	26	Abdomen	Liposuction	Not specified

AMSC = Adipose Tissue Derived Mesenchymal Stem Cell, BSA = Bovine Serum Albumin, CDU = Collagen Digestion Units, DPBS = Dulbecco’s Phosphate-Buffered Saline, DMEM = Dulbecco’s Modified Eagle Medium, EDTA = Ethylenediaminetetraacetic acid, FBS = Foetal Bovine Serum, MSC = Mesenchymal Stem Cell, PBS = Phosphate-Buffered Saline, SVF = Stromal Vascular fraction, USS = Ultrasound.

**Table 4:** Cellular characteristics for SVF studies, *n* = 7.

Authors	Number of Cells Used/Multiple Injections	Method of Delivery of Cells	SVF Pre-Treatment	Follow-Up Period (Weeks)	Harvest Site	Method of Harvest	MSC Surface Marker Validation Via Flow Cytometry
Garza et al. (2020) [69]	3.0 × 10^7^, 1.5 × 10^7^, 0 cells	Intra articular injection under USS guidance into unspecified joint space	SVF from dissociated tissue was centrifuged and the SVF cell pellet was extracted, resuspended for injection.	48	Abdomen	Liposuction	Not specified
Hong et al. (2019) [70]	7.45 × 10^6^ cells	Intra articular injection under arthroscopic guidance into unspecified location in joint space	The SVF from the lipoaspirate was isolated by means of collagenase digestion. The SVF was then washed twice with PBS to remove collagenase.	48	Abdomen	Liposuction	Not specified
Tran et al. (2019) [78]	9–12 × 10^7^ cells	Intra articular injection under arthroscopic guidance into chondral defect site	The SVF from the lipoaspirate was isolated through collagenase treatment. The SVF was then diluted with normal saline 0.9% to obtain 6 mL of solution containing 90–120 million cells to administer via injection.	96	Abdomen	Liposuction	Not specified
Yokota et al. (2017) [68]	Unknown, however estimated to be 3 × 10^7^ cells	Intra articular injection into unspecified location in joint space	Autologous SVF cells were collected in a sterile single-use functionally-closed system, requiring approximately 2–2.5 h.	4	Abdomen	Liposuction	Not specified
Nguyen et al. (2016) [67]	1 × 10^7^ cells	Intra articular injection under arthroscopic guidance into chondral defect site	The adipose tissue was digested using collagenase and centrifuged, the pellet was suspended in PBS for cell counting before injection.	72	Abdomen	Liposuction	Not specified
Koh et al. (2013) [66]	1.18 × 10^6^ cells	Intra articular injection into unspecified location in joint space	SVF was derived from fat pad tissue and mixed with 3.0 mL of platelet-rich plasma for injection.	97.2	Infrapatellar fat pad	Surgical excision of infrapatellar fat pad	Not specified
Panni et al. (2019) [74]	Not specified	Intra articular injection under arthroscopic guidance into unspecified location in joint space	The harvested fat was processed with the Lipogems^®^ ortho kit. The final product was transferred directly to syringes for injection.	61.2	Abdomen	Liposuction	Not specified

AMSC = Adipose Tissue Derived Mesenchymal Stem Cell, CDU = Collagen Digestion Units, DPBS = Dulbecco’s Phosphate-Buffered Saline, DMEM = Dulbecco’s Modified Eagle Medium, FBS = Foetal Bovine Serum, MSC = Mesenchymal Stem Cell, PBS = Phosphate-Buffered Saline, SVF = Stromal Vascular fraction, USS = Ultrasound.

**Table 5:** Outcomes and complications, *n* = 18.

Authors	Outcome Measures	Pre-Treatment WOMAC Scores	Post Treatment WOMAC Scores	Conclusions Based on Outcomes	Adverse Events	Nature of Complications
Lee et al. (2019) [64]	WOMAC, VAS, KOOS, ROM, K-L, Joint space width of medial and lateral compartment and HKA angle	Baseline WOMAC score was 60.0 (±17.0 SD)	At 6 months post procedure WOMAC scores were 26.7 (±13.3 SD)	Single injection of AD-MSCs led to a 55% reduction in the WOMAC total score, 59% in the pain score, 54% in the stiffness score, and 54% in the physical function score at 6 months. Significant improvements in the VAS, KOOS, ROM scores were also seen. K-L grade, joint space width of medial and lateral compartment, and HKA angle did not change significantly over 6 months in either groups. No evidence of significant cartilage regeneration in MRI at 6 months after the injection.	8AEs	6 cases of arthralgia and 2 cases of joint swelling after the procedure.
Freitag et al. (2019) [49]	NPRS-11, WOMAC, KOOS, MOAKS	WOMAC scores were 59.6 (±17.9 SD) for the one injection group and 54.4 (±18.2 SD) for the two-injection group	WOMAC scores were 84 (±9.4 SD) for the one injection group and 87.3 (±8 SD) for the two-injection group at 12 months.	NPRS-11 scores were greater when compared with baseline (< 0.05) throughout all time points in all treatment groups. There was no difference however between treatment groups. KOOS and WOMAC improved in all subscales during follow-up to 12 months. Two-thirds of the control group showed cartilage loss. 30% of the one-injection group had further cartilage loss, 50% had progression of osteophyte formation at 12 months. 89% in the two-injection group had either no progression or improvement in cartilage loss.	7 AEs and 1 SAE in the one injection group. 8AEs and 1 SAE in the first injection of the two-injection group. 10 AEs in the second injection in the two-injection group.	Mild AEs: minor discomfort, bruising and/or swelling after the injection. SAEs were classified as pain and sweeling for 4 weeks after injection which impacted the daily activities of life for the patient.
Song et al. (2018) [48]	WOMAC, NPRS-11, SF-36,	WOMAC scores were 34.75 (±17.05 SD) at baseline	WOMAC scores were 25.94 (±16.09 SD), 20.38 (±19.89 SD), 22.77 (±22.72 SD), 15.00 (±11.36 SD) and 12.44 (±8.99 SD) in the 12th, 24th, 48th, 72nd and 96th week.	WOMAC scores improved with time leading up to follow-up in all groups. Significant improvements in the NPRS-11 scores in the low- and high-dose groups were first observed at three months following treatment. A statistically significant reduction in SF-36 scores were only found in the 12th and 96th week of follow-up The volume of knee cartilage increased over the course of follow-up. This was more apparent in the high-dose group	8 AEs in the low dose group (66.67%). 7 AEs in the middle dose group (58.33%). 6 AEs in the high dose group (50%).	No SAEs or deaths. All complications were AEs. These were most commonly transient pain and swelling of joints, which were mild to moderate and were spontaneously relieved within 7 days without special treatment. One patient experienced mild oedema and cramps of bilateral lower extremities, which were relieved in 21 days without treatment and not related to the MSC treatment.
Roato et al. (2019) [73]	WOMAC, VAS, K-L	WOMAC score was 45.91 (±2.8) pre procedure. (NO SE OR SD GIVEN)	WOMAC scores were 27.47 (±3.02), 15.84 (±2.5) and 12.97 (±2.3) at 3 months, 6 months and 18 months post procedure. (NO SE OR SD GIVEN)	Significant improvement of VAS and WOMAC scores, with a significant pain reduction and increased mobility at 3, 6, and 12 months follow-up. No increase in the thickness of cartilage at 18 months.	1 SAE	Swelling persisted two months after surgery
Hudetz et al. (2019) [71]	KOOS, WOMAC, VAS	WOMAC baseline score was 55.38 (±18.8 SD)	WOMAC scores after 12 months was 32.25 (±14.6 SD)	All scores significantly improved after treatment.	0 AEs or SAEs	N/A
Spasovski et al. (2018) [65]	KSS, HSS, Lysholm score, VAS, MOCART	N/A	N/A	All outcomes significantly improved at 3 and 6 months. However, there was no further improvement beyond 12 or 18 months after treatment.	N/A	N/A
Jo et al. (2017) [62]	WOMAC, VAS, KSS, KOOS, K-L, Joint space width of the medial compartment, mechanical axis with weight bearing line, and anatomical axis	WOMAC scores were 43.3 (±12.7 SE) for the low dose group, 69.0 (±5.9 SE) for the mid dose group and 54.2 (±5.2 SE) for the high dose group.	WOMAC scores were 25.3 (±19.5 SE), 14.7 ± (12.7 SE) and 17.0 (±9.8 SE) at 6 months, 1 year and 2 years respectively for the low dose group. WOMAC scores were 48.5 (±9.5 SE), 13.1 (±10.0 SE) and 25.1 (±11.0 SE) at 6 months, 1 year and 2 years respectively for the middle dose group. WOMAC scores were 32.8 (±6.3 SE), 16.0 (±4.4 SE) and 19.0 (±5.5 SE) at 6 months, 1 year and 2 years respectively for the high dose group.	The WOMAC, VAS and KSS scores improved in the high-dose group at 6 months and 1 year. Non-significant trends in the low and middle dose groups. Significant improvement in KSS scores in the low dose groups up to one year. The sports subscore of the KOOS improved until 2 years for the high-dose group. No statistically significant improvements were found in the quality-of-life subscore of the KOOS for any of the dose groups.	None	None
Bansal et al. (2017) [77]	WOMAC, 6MWD, cartilage thickness	WOMAC score was 64 at baseline (NO SE OR SD GIVEN)	WOMAC scores were 52, 46, 42, 38 and 41 at 3 months, 6 months, 12 months, 18 months and 24 months respectively. (NO SE OR SD GIVEN)	Significant changes in the WOMAC and 6MWD scores were noted in both the subsets and the total after 2 years as compared to the baseline. MRI evaluation demonstrated that cartilage thickness improved.	1 AE	Pain and swelling which resolved.
Pers et al. (2016) [72]	WOMAC, VAS, PGA, SAS, KOOS, OARSI, SF-36	WOMAC scores were 63.2 (±4.1 SD) for the low dose group, 65.5 (±8.1 SD) for the mid dose group and 65.2 (±2.3 SD) for the high dose group.	WOMAC scores were 24.6 (±8.6 SD), 22.0 (±8.5 SD) and 30.1 (±8.9 SD) at 1 week, 3 months and 6 months respectively for the low dose group. WOMAC scores were 45.8 (±9.1 SD), 52.8 (±9.6 SD) and 42.6 (±9.1 SD) at 1 week, 3 months and 6 months respectively for the middle dose group. WOMAC scores were 61.1 (±15.3 SD), 38.4 (±16.0 SD) and 42.6 (±16.0 SD) at 1 week, 3 months and 6 months respectively for the high dose group.	Statistically significant improvements in WOMAC, VAS, KOOS and SAS scores were only found in the low dose group at 1 week, 3 months and 6 months. No improvements in the SF-36 in any groups.	1 SAE and 5 AEs.	The SAE was unstable angina pectoris without increased cardiac markers, which was reported in 1 patient 3 months after ASC injection. The patient’s risk factors included hypertension and hyperlipidemia. Five AEs reported by four patients. There was slight knee pain/joint effusion occurred during the first week after ASC injection that resolved with nonsteroidal anti-inflammatory drugs in three patients and spontaneously in one patient.
Jo et al. (2014) [63]	WOMAC, VAS, KSS, K-L, Joint space width of the medial compartment, mechanical axis with weight bearing line, and anatomical axis, ICRS	WOMAC scores were 43.3 (±12.7 SE) for the low dose group, 69.0 (±5.9 SE) for the mid dose group and 54.2 (±5.2 SE) for the high dose group.	WOMAC scores were 44.0 (±4.4 SE), 30.0 (±12.0 SE), 38.7 (±24.7 SE) and 25.3 (±19.5 SE) at 1, 2, 3 and 6 months respectively for the low dose group. WOMAC scores were 72.3 (±4.3 SE), 51.3 (±6.5 SE), 51.3 (±6.7 SE) and 48.5 (±11.0 SE) at 1, 2, 3 and 6 months respectively for the mid dose group. WOMAC scores were 45.5 (±4.5 SE), 40.1 (±6.0 SE), 37.0 (±6.8 SE) and 32.8 (±6.3 SE) at 1, 2, 3 and 6 months respectively for the high dose group.	Significant improvement of the WOMAC and VAS at 6 months compared with baseline in the high-dose groups. This was not seen in the other treatment groups. Knee subsection of KSS significantly increased in the low-dose and the high-dose groups, but improvements in the function subsection of seen in the low-dose group only. Other parameters did not change significantly at 6 months in any groups. The ICRS grade of the cartilage defect significantly improved in the medial femoral and tibial condyle in the high-dose group at second-look arthroscopy. No significant change was found in the lateral parts of the joint.	1 AE and 1 SAE in the low dose group (66.6%). 2 AEs in the mid dose group (66.6%). 5 AEs in the high dose group (41.66%).	In the low dose group, the AE was an individual case of nasopharyngitis, and the SAE was a urinary calculus. In the mid dose group AEs were individual cases of nasopharyngitis, arthralgia and chest pain. In the high dose group AEs were individual cases of nasopharyngitis, arthralgia, back pain, cough and hypertriglyceridemia.
Yokota et al. (2019) [75]	KOOS, VAS, OARSI, K-L	N/A	N/A	Change in KOOS symptoms occurred earlier in the AMSC group than the SVF group, with significant improvement detected at 3 months follow-up. The extent of VAS improvement after injection was greatest in patients with mildest. Patients in the AMSC group had a greater improvement in VAS than patients in the SVF group, regardless of the extent of OA at baseline. The proportion of patients who responded to treatment as determined by the OMERACT-OARSI responder criteria was greater in the AMSC group than the SVF.	3AEs in the ASC group and 26 AEs in the SVF group.	In the ASC group, there was 1 case of joint swelling after the injection and 2 cases of abdominal induration after harvest. These were all self-limiting. In the SVF group, there were 3 cases of joint swelling after the injection. There were 6 cases of abdominal pain, 5 cases of abdominal swelling and 12 cases of abdominal induration after harvest. These were all self-limiting.
Garza et al. (2020) [69]	WOMAC, OS	Baseline WOMAC scores were 49.3 for the placebo group, 56.2 for the low dose group and 47.1 for the high dose group (THIS WAS THE MEAN. NO SE OR SD WAS GIVEN) Median was 49.8 (37.4–57.0), 51.6 (46.3–62.3) and 49.8 (35.6–55.2) for the placebo, low dose and high dose groups.	WOMAC scores for the placebo was 26.0, 22.9, 37.2 and 41.9 at 6 weeks, 3 months, 6 months and 1 year respectively. Median values were 23.0(14.2–37.4), 20.0 (16.0–32.0), 30.2 (21.4–55.2), 41.0 (19.5–55.2). WOMAC scores for the low dose group was 24.8, 19.7, 23.7 and 21.8 at 6 weeks, 3 months, 6 months and 1 year respectively. Median values were 20.0 (10.7–37.4), 14.0 (5.3–35.6), 26.7 (8.9–32.0), 12.5 (7.1–35.6) WOMAC scores for the high dose group was 25.7, 26.5, 20.0 and 13.2 at 6 weeks, 3 months, 6 months and 1 year respectively. Median values were 27.0 (14.2–36.0), 27.0 (10.7–34.7), 8.9 (3.6–32.0), 3.6 (0.0–26.7)	All groups displayed a reduction in total WOMAC score from baseline at 6 month follow-up. All treated groups continued to demonstrate lower total WOMAC scores 1 year after injection as compared with baseline scores and sixth month scores. There was no change in cartilage thickness detected at six month follow-up.	0 AEs or SAEs	N/A
Hong et al. (2019) [70]	VAS, WOMAC, ROM, WORMS, MOCART	Baseline WOMAC pain score was 9.50 (±3.92 SD) for the control group and was 9.44 (±3.90 SD) for the treatment group. Baseline WOMAC stiffness score was 3.00 (±1.55 SD) for the control group and was 3.31 (±1.82 SD) for the treatment group.	WOMAC pain scores were 8.94 (±4.98 SD), 11.56 (±6.84 SD), 12.88 (±5.73 SD) and 15.19 (±4.29 SD) at 1 month, 3 months, 6 months and 12 months respectively for the control group. WOMAC stiffness scores were 4.38 (±2.22 SD), 4.94 (±2.49 SD), 5.44 (±2.56 SD) and 5.69 (±2.57 SD) at 1 month, 3 months, 6 months and 12 months respectively for the control group. WOMAC pain scores were 6.25 (±3.02 SD), 2.13 (±3.52 SD), 1.5 (±3.84 SD) and 1.44 (±4.77 SD) at 1 month, 3 months, 6 months and 12 months respectively for the treatment group. WOMAC stiffness scores were 1.75 (±1.59 SD), 1.12 (±1.80 SD), 0.81 (±1.59 SD) and 1.06 (±2.11 SD) at 1 month, 3 months, 6 months and 12 months respectively for the treatment group.	In the treated group, all scores including VAS, WOMAC pain, WOMAC stiffness, and knee ROM was founded to be significantly improved at one, three, six, and 12-months follow-up as compared with baseline scores within the treated groups and against control groups. Both WORMS and MOCART MRI scores showed a statistically significant improvement in the treatment group, while a deterioration in the control group.	10 AEs	4 AEs relating to abdominal pain after harvest which resolved after 1 week. 6 cases of pain and swelling in both knees after surgery. These all resolved after 2 weeks with analgesia.
Tran et al. (2019) [78]	VAS, WOMAC, OS, BME, K-L	WOMAC scores were 52.0 (±18.26 SD) and 42.64 (±12.51 SD) at baseline for patients with KL OA grade 2 and 3 respectively.	For KL OA grade 2 patients, WOMAC scores were 24.25 (±19.77 SD) and 18.25 (±20.07 SD) at 12 and 24 months respectively. For KL OA grade 3 patients, WOMAC scores were 18.21 (±8.20 SD) and 9.00 (±8.46 SD) at 12 and 24 months respectively.	No significant difference was found between the VAS scores of the treatment and placebo groups at 12 months.A decreasing trend in the VAS and WOMAC scores of the treatment group was observed up to 24 months compared to controls. Between 12 and 24 months, the VAS scores increased in the placebo group. MRI results showed that after 24 months of treatment, bone marrow oedema was decreased in both the placebo and the SVF treatment groups, the latter demonstrated a greater effect. The Outbridge score also decreased in the SVF-treated group.	N/A	N/A
Yokota et al. (2017) [68]	JKOM, WOMAC, VAS	Baseline WOMAC scores were 49.6 (±20.4 SD)	WOMAC scores were 43.0 (±17.4 SD) and 36.5 (±21.9 SD) at 1 month and 6 months after treatment respectively	JKOM, WOMAC, and VAS scores were significantly improved compared to baseline one month following treatment. This effect was also observed at the six-month visit. JKOM scores improved by an average of 35% over baseline compared to a 32% improvement in WOMAC, and 40% for VAS.	26 AEs	All patients experienced pain and swelling at the fat harvest and injection sites. These however resolved after a few days with analgesia.
Nguyen et al. (2016) [67]	WOMAC, Lysholm score, VAS, OS, BME, JMA,	WOMAC scores for the placebo group was 47.27 (±17.13 SD). WOMAC scores for the treatment group was 42.87 (±16.29 SD)	WOMAC scores for the placebo group was 23.27 (±15.61 SD) and 25.60 (±19.69 SD) at 6 and 12 months respectively. WOMAC scores for the treatment group were 19.27 (±14.87 SD) and 17.33 (±14.91 SD) at 6 and 12 months respectively.	WOMAC scores significantly decreased compared with baseline scores at 6 and 12 months.WOMAC scores between the treatment and placebo groups were not significantly different at 12 months, but a significant difference was seen at 18 months. VAS and Lysholm scores improved in the treatment group compared to pre treatment scores at all follow-up timepoints.	0 AEs or SAEs	N/A
Koh et al. (2013) [66]	WOMAC, lysholm score, VAS, WORMS	Baseline WOMAC score was 49.9 (±12.6 SD)	After final follow-up post procedure WOMAC scores were 30.3 (±9.2 SD)	WOMAC scores decreased in the treatment group over the follow-up period. Greater changes in WOMAC score were seen in subjects injected with greater cell numbers. Lysholm and VAS scores also significantly improved over the follow-up period. Significant reduction was observed in the WORMS cartilage subscore.	1 AE	Notable pain and swelling after injection for 2 weeks. This was self-limiting.
Panni et al. (2019) [74]	IKS, VAS,	N/A	N/A	96.2% of treated subjects reported improvements in knee function and/or pain. A subset (62%) achieved complete or near-complete function recovery and/or pain relief. Two (3.9%) patients reported slight reduction of the pain.	3AEs	Transient haematoma after harvest

6MWD = 6 Minute Walk Distance, BMA = Bone Marrow Oedema, HKA = hip-knee-ankle, HSS = Hospital for Special Surgery Knee score, ICRS = International Cartilage Repair Society, IKS = International Knee Society, JKOM = Japanese Knee Osteoarthritis Measure, JMA = Joint Motion Amplitude, KOOS = Knee Injury and Osteoarthritis Outcome Score, K-L = Kellgren-Lawrence, KSS = Knee Society Score, MOAKs = MRI Osteoarthritis Knee Score, MOCART = 2D Magnetic Resonance Observation of Cartilage Repair Tissue, NPRS = Numeric Pain Rating Scale, OARSI = Osteoarthritis Research Society International, OS = Outerbridge Classification System, PGA = Patient Global Assessment score, ROM = range of motion, SAS = Short Arthritis Assessment scale, SF-36 = Short Form-36, WOMAC = Western Ontario and McMaster Universities Osteoarthritis Index, WORMS = Whole-Organ MRI Score, VAS = visual analogue scale.

## Data Availability

Not Applicable.

## Supplementary Materials

The following are available online at https://www.mdpi.com/article/10.3390/cells10061365/s1, Table S1 Critical appraisal of the non-randomised studies included in this systematic review, using the ROBINS-1 tool *n* = 13, Table S2. Critical appraisal of the randomised studies included in this systematic review, using the RoB-2 tool *n* = 5

## Appendix A

**Databases searched:**

OVID MEDLINE^®^: 1946 TO JUNE WEEK 4 2020

Date of search: 29/06/20

Date range searched: January 1946 to June 2020

**S**EARCH STRATEGY

1. exp cartilage/ or exp “bone and bones”/
2. exp Injections, Intra-Articular/
3. exp Osteoarthritis, Hip/ or exp Osteoarthritis/ or exp Osteoarthritis, Spine/ or exp Osteoarthritis, Knee/
4. exp Mesenchymal Stem Cells/ or exp Mesenchymal Stem Cell Transplantation/
5. exp Transplantation, Homologous/ or exp Stem Cell Transplantation/ or exp Transplantation, Autologous/ or exp Mesenchymal Stem Cell Transplantation/
6. exp Adipose Tissue/ or adipose.mp.
7. adipo*.tw.
8. exp Autografts/
9. exp Heterografts/
10. 4 or 5 or 8 or 9
11. 1 or 2 or 10
12. 6 or 7
13. 11 and 12
14. 3 and 13
15. Limit 14 to (English language and humans)

**EMBASE:** 1974 TO 2019 JUNE 26

Date of search: 29/06/20

Date range searched: January 1974 to June 2019

**S**EARCH STRATEGY

1. exp cartilage/
2. exp intraarticular drug administration/
3. exp knee osteoarthritis/
4. exp mesenchymal stem cell transplantation/ or exp mesenchymal stem cell/
5. exp autograft/
6. exp adipose tissue/
7. adipo*.tw.
8. 4 or 5
9. 1 or 2 or 8
10. 6 or 7
11. 9 and 10
12. 3 and 11
13. Limit 12 to (human and English language)

**COCHRANE LIBRARY:** 1946 TO JUNE 2020

Date of search: 29/06/20

Date range searched: January 1946 to June 2020

**SEARCH STRATEGY**

**#1 MeSH descriptor:** [Cartilage] explode all trees

#2 MeSH descriptor: [Injections] explode all trees

#3 MeSH descriptor: [Osteoarthritis] explode all trees

#4 MeSH descriptor: [Mesenchymal Stem Cells] explode all trees

#5 MeSH descriptor: [Stem Cells] explode all trees

#6 MeSH descriptor: [Adipose Tissue] explode all trees

#7 MeSH descriptor: [Autografts] in all MeSH products

#8 MeSH descriptor: [Heterografts] in all MeSH products

#9 #1 or #2 or #3

#10 #4 or #5 or #6 or #7 or #8

#11 #10 and #9

#12 #11 and #6

WEB OF SCIENCE: 1900 TO 2020

Date of search: 29/06/20

Date range searched: 1900 to June 2020

**SEARCH STRATEGY**

**#1:** (TS = (cartilage or inject*)) Indexes=SCI-EXPANDED, CPCI-S Timespan=1900–2020

#2: (TS=(osteoarthritis)) Indexes=SCI-EXPANDED, CPCI-S Timespan=1900–2020

#3: (TS=(mesenchymal stem cell or stem cell or homologous or autologous or autograft or heterograft)) Indexes=SCI-EXPANDED, CPCI-S Timespan=1900–2020

#4: (TS=(adipose)) Indexes=SCI-EXPANDED, CPCI-S Timespan=1900–2020

#5: #3 or #1 Indexes=SCI-EXPANDED, CPCI-S Timespan=1900–2020

#6: #5 and #2 Indexes=SCI-EXPANDED, CPCI-S Timespan=1900–2020

#7: #6 and #4 Indexes=SCI-EXPANDED, CPCI-S Timespan=1900–2020

**CLINICAL TRIALS.GOV:** 1900 TO 2020

Date of search: 29/06/20

Date range searched: 1900to July 2019

#1 Osteoarthritis, knee

#2 Adipose
