# Supplementary material for: Meta-Analysis of Adipose Tissue Derived Cell-Based Therapy for the Treatment of Knee Osteoarthritis
Source: Cells. 2021 Jun 1;10(6):1365. doi: 10.3390/cells10061365 (PMC8228374; doi:10.3390/cells10061365)
Supplement: Supplementary file 1 [file cells-10-01365-s001.zip › cells-1200893-supplementary.pdf]

Supplementary Table 1: Critical appraisal of the non-randomised studies included in this systematic review, using the ROBINS-1 tool n = 13.

**Paper**	**Bias due to confounding**	**Bias in selection of participants into the study**	**Bias in classification of interventions**	**Bias due to deviations from intended interventions**	**Bias due to missing data**	**Bias in measurement of outcomes**	**Bias in selection of the reported result**	**Overall bias**
Jo et al (2014) [57]	Low risk	Low risk	Low risk	Low risk	Low risk	Low risk	Low risk	Low risk
Jo et al (2017) [56]	Low risk	Low risk	Low risk	Low risk	Low risk	Low risk	Low risk	Low risk
Pers et al (2016) [64]	Low risk	Low risk	Low risk	Low risk	Low risk	Low risk	Low risk	Low risk
Roato et al (2019) [66]	Low risk	Low risk	Low risk	Low risk	Low risk	Moderate risk	Low risk	Low risk
Koh et al (2013) [69]	Low risk	Low risk	Low risk	Low risk	Low risk	Moderate risk	Low risk	Low risk
Panni et al (2019) [71]	Low risk	Low risk	Low risk	Low risk	Moderate risk	Moderate risk	Low risk	Low risk
Yokota et al (2019) [72]	Low risk	Low risk	Low risk	Low risk	Low risk	Low risk	Low risk	Low risk
Spasovski et al (2018) [68]	Low risk	Low risk	Low risk	Low risk	Low risk	Moderate risk	Low risk	Low risk
Nguyen et al (2016) [70]	Low risk	Low risk	Low risk	Low risk	Low risk	Moderate risk	Low risk	Low risk
Yokota et al (2017) [58]	Low risk	Low risk	Low risk	Low risk	Low risk	Moderate risk	Low risk	Low risk
Bansal et al (2017) [60]	Low risk	Low risk	Low risk	Low risk	Low risk	Moderate risk	Low risk	Low risk
Tran et al (2019) [61]	Low risk	Low risk	Low risk	Low risk	Low risk	Moderate risk	Low risk	Low risk
Hudetz et al (2019) [63]	Low risk	Low risk	Low risk	Low risk	Low risk	Moderate risk	Low risk	Low risk

Supplementary Table 2: Critical appraisal of the randomised studies included in this systematic review, using the RoB-2 tool n = 5

Papers	1.0 algorithm result	2.0 algorithm result	3.0 algorithm result	4.0 algorithm result	5.0 algorithm result	Algorithm's overall judgement
Song et al (2018) [38]	Low	Low	Low	Low	Low	Low
Freitag et al (2019) [65]	Some	Some	Low	Low	Low	Low
Lee et al (2019) [67]	Low	Low	Low	Low	Low	Low
Garza et al (2020) [59]	Low	Low	Low	Low	Low	Low
Hong et al (2019) [62]	Low	Some	Low	Low	Low	Low
